# Insights into Molecular Features of *Venerupis decussata* Oocytes: A Microarray-Based Study

**DOI:** 10.1371/journal.pone.0113925

**Published:** 2014-12-03

**Authors:** Marianna Pauletto, Massimo Milan, Joana Teixeira de Sousa, Arnaud Huvet, Sandra Joaquim, Domitília Matias, Alexandra Leitão, Tomaso Patarnello, Luca Bargelloni

**Affiliations:** 1 Department of Comparative Biomedicine and Food Science, University of Padova, Legnaro, Italy; 2 IFREMER, Institut Français de Recherche pour l’Exploitation de la Mer, Laboratoire des Sciences de l’Environnement Marin, Plouzané, France; 3 IPMA, Instituto Português do Mar e da Atmosfera, Olhão, Portugal; 4 CIIMAR, Interdisciplinary Centre of Marine and Environmental Research, University of Porto, Porto, Portugal; 5 Environmental Studies Center, Qatar University, Doha, Qatar; Universitat de Barcelona, Spain

## Abstract

The production of *Venerupis decussata* relies on wild seed collection, which has been recently compromised due to recruitment failure and severe mortalities. To address this issue and provide an alternative source of seed, artificial spawning and larval rearing programs were developed. However, hatchery-based seed production is a relatively new industry and it is still underdeveloped. A major hurdle in the European clam seed production is the control of spawning and reproduction, which is further hindered by the impossibility of obtaining fertile gametes by gonadal “stripping”, as meiosis re-initiation is constrained to a maturation process along the genital ducts. In the present study, oocytes were collected from 15 females and microarray analyses was performed to investigate gene expression profiles characterizing released and stripped ovarian oocytes. A total of 198 differentially expressed transcripts between stripped and spawned oocytes were detected. Functional analysis carried out on these transcripts highlighted the importance of a few biological processes, which are most probably implicated in the control of oocyte competence. Significant differences were observed for transcripts encoding proteins involved in meiosis progression (e.g. dual specificity phosphatase CDC25), WNT signalling (e.g. frizzled class receptor 8, wingless-type MMTV integration site family member 4), steroid synthesis (e.g. progestin and adipoQ receptor family member 3, cytochrome P450-C17), mRNA processing (e.g. zinc finger protein XlCOF28), calcium regulation (e.g. regucalcin, calmodulin) and ceramide metabolism (ceramidase B, sphingomyelinase). This study provides new information on transcriptional profiles putatively associated with ovarian egg infertility, and suggests potential mechanisms regulating early oocyte development in clams. Genes which were differentially expressed between stripped and spawned oocytes might have a pivotal role during maturation process in the gonadal duct and could be interesting targets for further functional studies aiming to make ovarian oocytes fertilizable.

## Introduction

The grooved carpet shell *Venerupis decussata* is a native European bivalve species and, although its global aquaculture production is still relatively low in Europe (4.137 tons in 2011) [1], it has a high economic value. *V. decussata* production is economically important in many Mediterranean countries, mainly Portugal, Italy and Spain. However, due to the difficulties in broodstock conditioning and larval rearing [2] the culture of this species relies mainly on natural recruitment of seed, it is therefore limited by its availability and would greatly benefit from hatchery-produced spat.

Among the major hurdles reported in hatchery production of this species, spawning control and gamete quality are the most important issues. Notably, spawning success in the European clam is not predictable, with frequent failures to induce gametes emission. Moreover, this cannot be overcome by stripping, a practice for collecting oocytes before egg emission, widely used in some bivalve species (*e.g*. in cupped oysters), where stripped eggs can be fertilized. The impossibility to obtain fertile gametes by gonadal stripping in *V. decussata* clearly suggests the existence of a maturation process along the genital ducts. Indeed, meiotic progression in germ cells is not regulated in the same manner across molluscan species. While full-grown oocytes of all bivalves are blocked in ovaries at prophase I stage, some crucial differences are observed in spawned eggs. In bivalves such as *Spisula* or *Barnea* spawned oocytes are arrested at prophase I and fertilization occurs at this stage leading to meiosis re-initiation [3]–[5]. In contrast, bivalves such as *Venerupis* and *Crassostrea* [6] exit from prophase I and undergo germinal vesicle breakdown (GVBD) after spawning and then are further blocked at the first metaphase (metaphase I). The release from metaphase I is naturally triggered by fertilization or can be artificially induced. However in both cases, it seems that an increase in intracellular [Ca2+] has a pivotal role in meiosis re-initiation [7]–[9]. Although both *Venerupis* and *Crassostrea* oocytes encounter two blockages during meiosis I, their meiotic progression is not regulated in the same way. Naturally spawned oyster oocytes, like in *Venerupis*, are blocked at metaphase I and wait for fertilization to re-enter meiosis. Although oyster oocytes isolated from ovaries (stripped) are still at prophase I, their suspension in seawater permits GVBD and progression up to metaphase I, thus allowing fertilization by sperm [10]. On the opposite, stripped and hydrated oocytes from *Venerupis* remain blocked at prophase (prior to GVBD) and cannot be fertilized. The molecular determinants of this crucial difference are still unknown.

To date, the mechanisms controlling oocyte maturation in *V. decussata* have been scarcely studied [2]. Conversely, in other bivalves meiosis in female gametes was extensively analysed and a few major factors regulating oocyte maturation processes were identified. Notably, it was demonstrated that serotonin (5-HT), thought to be the natural inducer of oocyte maturation in bivalves [11], triggers germinal vesicle breakdown (GVBD) in vitro when added to *Spisula*, *Barnea*, *Venerupis philippinarum* or *Crassostrea* isolated prophase I oocytes [6], [8], [10], [12]–[15]. Moreover, it has been suggested that in *V. philippinarum*, the mechanisms by which 5-HT promotes GVBD involve an increase in intracellular [Ca2+], which is thought to be mediated in turn by inositol 1,4,5-trisphosphate receptors (IP3r) and specific 5-HT receptors [8], [16]–[17]. Despite these studies pointed out a few interesting factors driving meiosis progression in bivalves, little is known on cell signalling during gamete maturation and spawning induction in these species. Up to date, only few gene expression and proteomic studies have been carried out in bivalve oocytes mainly for quality purpose e.g. [18]–[19] and a comprehensive picture of the molecular processes characterizing their maturation is still lacking. Remarkably, when talking about gene expression and mRNA in oocytes, an interesting point should be taken into account. At prophase I, immature oocytes show a prominent nucleus (the germinal vesicle), which contains de-condensed chromatin [20], thus oocytes at this stage are transcriptionally active until meiosis resumption, when transcription is generally thought to cease [21]. However, translation of the stored pool of mRNAs continues throughout the final stages of meiosis [22] to synthetize proteins that are crucial for supporting not only oocyte maturation (meiotic maturation), but also the phase prior to zygote-embryonic genome activation [23]–[25].

Beside gamete maturation, several factors are still limiting hatchery production of *V. decussata*. The absence of established methods for larval rearing, a scarce knowledge on the best hatching practices to improve metamorphosis synchronization and settlement are only a few additional issues about European clam seed production which is an activity far from being completely technically controlled. In this context, the improvement of knowledge on broodstock management and gamete maturation processes is a key step to improve seed production of this emerging bivalve species.

In the present study, we sampled 15 females in the main production area of *V. decussata* in Portugal, Ria de Aveiro (Western coast of Portugal). For 10 of them, mature oocytes were collected by spawning induction whereas oocytes from the five remaining females were collected through gamete stripping. Microarray analysis was performed on these samples by using a custom oligonucleotide microarray containing 51,678 probes representing unique contigs described and used in *de Sousa* et al. [26]. The main objective of the present work was to investigate gene expression profiles characterizing released oocytes and ovarian oocytes obtained by stripping providing new information on transcriptional profiles putatively associated with ovarian egg infertility.

## Methods

### Ethics statement

The European clam is not considered as an endangered or protected species in any Portuguese or international species catalogue, including the CITES list (www.cites.org). The European clams from Ria de Aveiro (40°42′N 08°40′W) were produced and captured with the permission of DGRM (Direçaõ-Geral de Recursos Naturais, Segurança e Serviços Marítimos) and APA (Agencia Portuguesa do Ambiente).

### Biological samples and RNA isolation

Clams were sampled in Ria de Aveiro (Western coast of Portugal) and conditioned in common garden from May 2013 to June 2013 (one month) in the experimental bivalve hatchery of the Portuguese Institute of Sea and Atmosphere (IPMA) in Tavira, Portugal, to accelerate their gonad development under common rearing facilities. Food regime consisted of a mix diet of three microalgae, 1/3 *Isochrysis galbana* (clone T-ISO), 1/3 *Skelectonema costatum* (Ria Formosa autochthones clone) and 1/3 *Chaetoceros calcitrans*.

Released oocytes were obtained by thermal stimulation to induce spawning of females, consisting on exposure to alternate cycles of 29°C (1 hour) and 5°C (30 minutes) [27]. As each female begun to spawn, it was removed from the spawning tank and transferred to an individual spawning beaker with filtered seawater at the same temperature [27]. Once spawning was completed, the obtained oocytes were gently washed into a clean glass. From each female, 20 000 oocytes were taken for preservation. The remained oocytes of each female spawning were mixed with a sperm suspension (from 7 males) by gentle agitation, aiming at obtaining around 10 spermatozoids by oocyte in a microscopic view [27]–[28]. Moreover, the D-larval rate (ratio between the number of D-larvae at 48 h post fertilization and the number of incubated eggs) of each eggs batch was registered.

In addition, gonads from five females were dissected and oocytes were collected through a practice known as “gamete stripping”. As the name indicates, this procedure involves removal of gametes from gonad tissue. Briefly, fully ripe gonads were slashed repeatedly with a scalpel and washed with filtered seawater to harvest the gametes.

The spawned/stripped oocytes were collected and filtered in a 40 µm sieve. The oocytes were transferred into an Eppendorf tube and, after a short spin, the seawater was removed. To remove salts, the pellet of oocytes was re-suspended with a solution of ammonium formate (3% w/v), which was immediately removed after a short spin. Then the oocytes were included in 1,5 ml of Extract all solution (Eurobio) and preserved in liquid nitrogen until RNA isolation.

RNA was purified by following the manufacturer instructions and a DNAse treatment was carried out through RTS DNAse Kit (MO-BIO). Samples concentration was measured in a NanoDrop ND-1000 spectrophotometer and the RNA quality was assessed through the Bioanalyzer 2010 instrument (Agilent).

### Labeling and microarray hybridization

Microarray experiments were performed by using an Agilent microarray platform obtained in a previous study [26], which included a total of 59,951 probes, 85.7% out of them were annotated by blastx (cut off e-value of <1.0 E-5) searches against high quality proteins of UniProtKB/SwissProt (release 2013_07 - June 26, 2013), *Danio rerio*, *Gasterosteus aculeatus, Drosophila melanogaster, Homo sapiens, Strongylocentrotus purpuratus, Ciona intestinalis, Caenorhabditis elegans* and *Lottia gigantea* available on Ensembl Genome Browser (release 72, June 2013), *Crassostrea gigas* (http://oysterdb.cn/) [29]. and *Daphnia pulex* v1 (http://genome.jgi-psf.org/Dappu1/Dappu1.home.html). Probe sequences and other details on the microarray platform can be found in the GEO database (http://www.ncbi.nlm.nih.gov/geo/) under accession number GPL17766.

Microarray experiments were carried out on a total of 15 samples corresponding to unfertile stripped oocytes and spawned oocytes. Sample labeling and hybridization were performed according to the Agilent One-Color Microarray-Based Gene Expression Analysis protocol with the Low Input Quick Amp Labeling kit. Refer to *de Sousa* et al. [26] for details about labeling and hybridization procedures.

### Data acquisition, correction and normalization

Hybridized slides were scanned at 2 µm resolution using an Agilent G2565BA DNA microarray scanner. Each slide was scanned two times at two different sensitivity levels: XDR Hi 100% and XDR Lo 10%. The two generated images were analysed together, data were extracted and background subtracted using the standard procedures provided in the Agilent Feature Extraction (FE) Software version 10.7.3.1. To evaluate goodness and reliability of spot intensity estimates the software returns a series of spot quality measures. All control features (positive, negative, etc.), except for Spike-in (Spike-in Viral RNAs), were excluded from subsequent analyses.

The fluorescence values were normalized by performing a quantile normalization in R statistical software. Statistical analyses were performed on 31,862 out of 59,951 probes with signal higher than background in at least 5 out of 15 target samples. A log base 2 transformation was applied to all expression values and finally the parametric Combat algorithm [30] was implemented in R in order to adjust for the known between-experiments batch effect (i.e. different microarray slides). Normalized data were deposited in GEO archive under accession number GSE58906.

### Data analysis

A Principal Component Analysis (PCA), using the TMeV 4.5.1 (TIGR MULTIEXPERIMENT VIEWER) [31] was applied, to assess the distribution of the studied groups. In addition, in order to find out the probes which most affected the Principal Component 1 (PC1) and Principal Component 2 (PC2) variance, the eigenvector values of each component were retrieved. To our knowledge, a generally accepted threshold to retain the most significant eigenvectors does not exist, thus for both PCs the whole set of probes was sorted in descending order by the absolute eigenvector values, and the first 5% of probes was arbitrary selected (a total of 1,593 probes).

Statistical tests implemented in the program Significance Analysis of Microarray (SAM) were used to identify differentially expressed probes between the stripped oocytes and the t spawned oocytes.

Only the differentially expressed probes showing a significant variation (False Discovery Rate (FDR) <1.5%; Fold change (FC)>1.5) have been selected.

A more systematic, functional interpretation of significant genes was then obtained through enrichment analysis using the Database for Annotation, Visualization, and Integrated Discovery (DAVID) software [32]. “KEGG Pathway”, “Biological process” (BP), “Molecular function” (MF) and “Cellular component” (CC) annotation categories were used by setting the gene count equal to 3 and the ease value equal to 0.1. Because DAVID database contains functional annotation data for a limited number of species, it was necessary to link the *V. decussata* transcripts with sequence identifiers that could be recognized in DAVID. This process was accomplished using UniProtKB/SwissProt feature identifiers corresponding to each probe. These identifiers were used to define a “gene list” and a “background” in the bioinformatic tool DAVID, corresponding to differentially transcribed clam genes and to all the transcripts that were represented on the array, respectively.

## Results

### Principal component analysis

**Figure 1:**
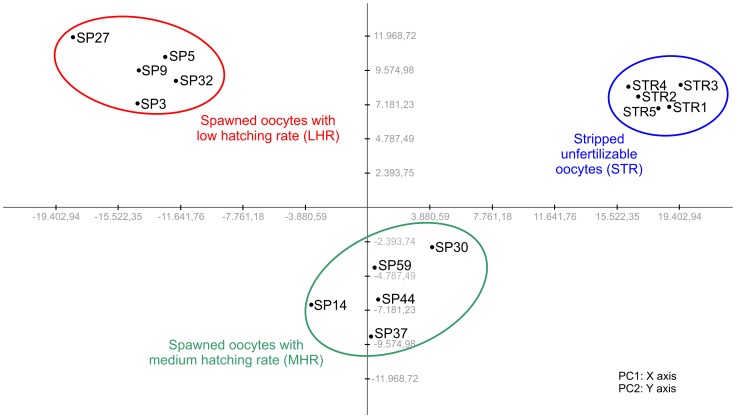
Principal Component Analysis. Spawned and stripped oocytes are identified by prefixes SP and ST, respectively. Different circles highlight the three groups considered for further analysis. Principal Component 1 (PC1) and Principal Component 2 (PC2) correspond to X axis and Y axis, respectively.

A Principal Component Analysis (PCA) was applied to the selected gene expression dataset (31,862 probes, see Methods) for the 15 oocytes samples. As shown in Figure 1, a distinct clustering of three different groups of samples was observed. The group of spawned oocytes was clearly separated into 2 sub-groups, which also differ from their D larval rates, defined and already employed successfully as a good proxy of oocyte quality [19]. Therefore we then considered three groups for further analysis: stripped oocytes (STR), spawned oocytes with low hatching rate (LHR, 5%–21% of D-larval rate) and spawned oocytes with medium hatching rate (MHR, 40%–47% of D-larval rate). STR, LHR and MHR eggs were clearly separated along the Principal Component 1 (x axis), which explained 29% of the variation. The expression profiles were also separated along the Principal Component 2 (y axis, 11% of the variation), but in this case STR and LHR oocytes did not show a marked divergence in expression patterns, while the separation of MHR eggs was remarkable. Notably, the expression patterns of the five stripped oocytes appeared to be similar, while spawned eggs of the two groups seemed less homogeneous.

Finally, the PCA outcome allowed the identification of the probes which maximally contributed to the PC1 or PC2 variance. Among the 1,593 probes (5% out of the total amount of probes) with the highest PC1 eigenvector values, 990 were annotated against the UniProtKB/SwissProt database and represented 852 unique genes. With regards to the PC2, 925 out of the 1,593 probes with the highest load were annotated and a total of 826 unique genes were recovered. The lists of probes with the highest PC1 and PC2 eigenvector values were reported in Table S1, together with their putative annotation against UniProtKB/SwissProt database.

### Comparison between spawned and stripped oocytes

**Figure 2:**
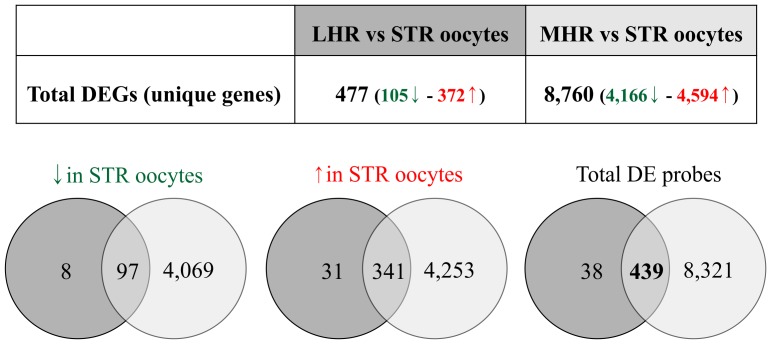
Differential expression analysis. Number of Differentially Expressed (DE) probes in the two comparisons (LHR vs STR in dark grey; MHR vs STR in light grey), determined through a two-class unpaired SAM. Arrows specify the way in which mRNA expression is different: green arrow “↓”and red arrow “↑” mean lower and higher expression in STR oocytes, respectively. Venn diagrams show the number of DE probes shared between the two comparisons. STR: stripped oocytes; LHR: low hatching rate oocytes; MHR: medium hatching rate oocytes.

In order to compare gene expression profiles between *V. decussata* oocytes, a two-class unpaired SAM test was carried out (FDR<1.5%; FC>1.5). Only the differentially expressed probes showing a significant variation (False Discovery Rate (FDR) <1.5%; Fold change (FC)>1.5) in both comparisons (LHR vs STR; MHR vs STR) have been selected. The number of significant probes obtained for each comparison and those that were significant in both analyses are summarized in Figure 2. The detailed lists of significant probes in each analysis are reported in Table S2. Since the goal of this study was the analysis of molecular signatures characterizing stripped and spawned oocytes, rather than quality of spawned oocytes (see discussion), the comparison between LHR and MHR oocytes was not carried out.

A comparison between significant probes in the two analyses allowed us the identification of a set of 439 transcripts (1.4% of all probes) that were differentially expressed between stripped and spawned oocytes, irrespective of D-larval rate. Except for one probe (lacking putative annotation), which showed an opposite trend of expression in the two comparisons (expression higher in STR than in LHR and lower in STR than in MHR), all significant variations reported for the remaining 438 probes had concordant direction (Figure 2). A putative UniProtKB/SwissProt accession ID was obtained for 235 out of 439 probes, corresponding to 198 unique genes (see Table S2), which were differentially expressed between the two oocyte conditions (stripped and spawned). Among the shared Differentially Expressed Genes (DEGs), important transcripts encoding regulators of sex steroids synthesis and activity, such as a progestin and adipoQ receptor family member 3 (PAQR3) and steroid 17-alpha-hydroxylase/17,20 lyase (CYP450-C17), were expressed at a higher level in the stripped oocytes. Interestingly, also a putative vitellogenin (*VG*) was expressed only in released oocytes. Moreover, gene expression of enzymes involved in the metabolism of ceramide showed significant variation. Sphingomyelin phosphodiesterase and neutral ceramidase B were expressed at a higher level in stripped oocytes, while a putative sphingomyelinase transcript was more abundant in spawned oocytes. A further interesting result was observed also for putative translin-associated protein X and oocyte zinc finger protein XlCOF28, whose mRNA level was higher and lower in ovarian oocytes, respectively. In addition, the mRNA levels of nucleoporin 37 (*NUP37*) and U8 snoRNA-decapping enzyme, both involved in mRNAs processing, were higher in stripped than in released oocytes.

Putative homologs of *C. gigas* calcium-activated chloride channel regulator 4 (two probes), regucalcin, and calmodulin were more abundant in stripped oocytes, while sodium/calcium exchanger 3 (*NCX3*) had a higher expression level in spawned oocytes.

As expected, mRNA profiles of genes regulating cell cycle progression were subjected to great variation. Notably, five probes encoding CDC25 (M-phase inducer phosphatases - MPIP) and a MPIP-like protein were expressed at a higher extent in released oocytes.

**Figure 3:**
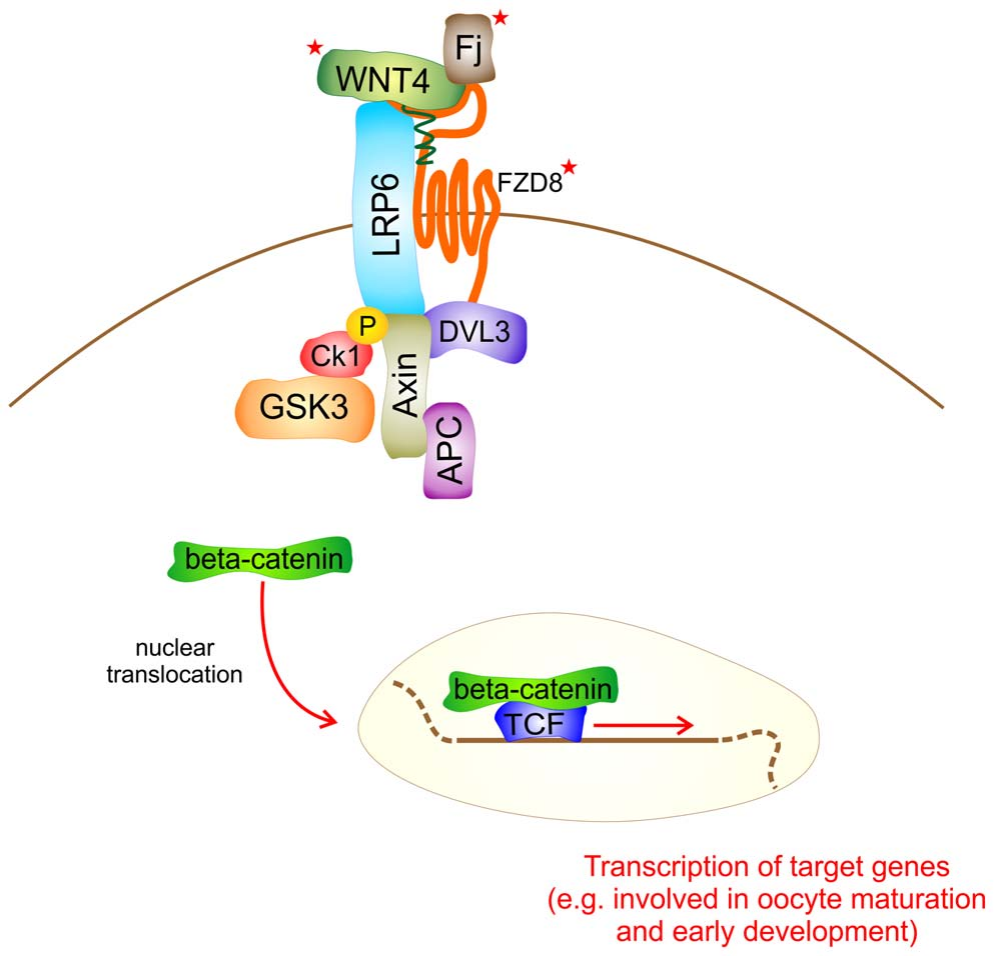
WNT signaling pathway. Binding of WNT4 to the receptor FZD8 and LRP6 leads to inhibition of beta-catenin degradation. Beta-catenin in turn interacts with members of the TCF/Lef-1 family of transcription factors to co-activate target gene transcription. WNT components whose mRNA was more abundant in clam released than in stripped oocytes are marked with a red star next to the gene symbol. LRP6: low density lipoprotein receptor-related protein 6; CK1: casein kinase 1; GSK3: glycogen synthase kinase 3; APC: adenomatous polyposis coli; TCF: transcription factor.

Finally, differential expression analysis also highlighted the importance of WNT signalling pathway. Frizzled 8 (*FZD8*), four-jointed protein (*FJ*) and wingless-type MMTV integration site family member 4 (*WNT4*) transcripts were found to be more abundant in released oocytes, suggesting a significant over-representation of WNT signal activities in female gametes after spawning. Figure 3 reports a schematic view of the WNT pathway (as known in *Drosophila*) and highlights the putative components whose mRNA was expressed at higher extent in *V. decussata* released oocytes.

In order to obtain a global picture that describes the main molecular pathways differentiating stripped and spawned oocytes, all putative annotated DEGs were used to define a gene list for functional annotation with DAVID. Enrichment analysis showed 7 CC terms, 16 BP terms, 9 MF terms and 1 KEGG to be significantly over-represented (Table 1). The only significant KEGG pathway was “ribosome” (dme03010), represented by three transcripts, more abundant in the stripped oocytes, 60S ribosomal protein L3, 60S acidic ribosomal protein P1 and 40S ribosomal protein S9. “Mitotic cell cycle” (GO:0000278), “translation” (GO:0006412), “WNT receptor signaling pathway” (GO:0016055) and “dephosphorylation” (GO:0016311) were the most represented among enriched BP terms with a fold enrichment (FE)>2. With regard to CC terms, one of the most represented ones was “ribonucleoprotein complex” (GO:0030529), which in oocytes is most likely involved in the storage and compartmentalization of incompletely polyadenylated mRNAs. Significant MF terms concerned activities that are classically involved in cell cycle regulation such as peptidase (GO:0008233) and phosphatase (GO:0016791) activities, and molecular functions that contribute to structural integrity like “structural molecule activity” (GO:0005198) and “structural constituent of ribosome” (GO:0003735).

**Table 1:** Enrichment analysis.

BP terms	Count	P-val	FE
GO:0016055∼Wnt receptor signaling pathway	6	0	6.02
GO:0009310∼amine catabolic process	5	0.01	5.98
GO:0006470∼protein amino acid dephosphorylation	6	0.01	4.1
GO:0016311∼dephosphorylation	6	0.03	3.55
GO:0006414∼translational elongation	4	0.03	5.78
GO:0046395∼carboxylic acid catabolic process	5	0.03	4.09
GO:0016054∼organic acid catabolic process	5	0.03	4.09
GO:0009063∼cellular amino acid catabolic process	4	0.04	5.29
GO:0051329∼interphase of mitotic cell cycle	4	0.04	5.29
GO:0051325∼interphase	4	0.04	5.18
GO:0051187∼cofactor catabolic process	3	0.05	8.11
GO:0006412∼translation	8	0.05	2.32
GO:0000278∼mitotic cell cycle	8	0.08	2.14
GO:0000279∼M phase	8	0.09	2.07
GO:0000226∼microtubule cytoskeleton organization	5	0.09	2.93
GO:0009066∼aspartate family amino acid metabolic process	3	0.1	5.65
**CC terms**	**Count**	**P-val**	**FE**
GO:0022626∼cytosolic ribosome	4	0	11.18
GO:0005576∼extracellular region	15	0.01	2.13
GO:0005840∼ribosome	7	0.01	3.54
GO:0044445∼cytosolic part	5	0.02	4.51
GO:0044421∼extracellular region part	8	0.04	2.43
GO:0033279∼ribosomal subunit	4	0.05	4.89
GO:0030529∼ribonucleoprotein complex	10	0.09	1.82
**MF terms**	**Count**	**P-val**	**FE**
GO:0005198∼structural molecule activity	12	0	3.06
GO:0003735∼structural constituent of ribosome	7	0	4.91
GO:0004725∼protein tyrosine phosphatase activity	6	0.01	4.93
GO:0004721∼phosphoprotein phosphatase activity	7	0.01	3.64
GO:0070011∼peptidase activity, acting on L-amino acid peptides	10	0.04	2.15
GO:0016831∼carboxy-lyase activity	3	0.04	8.92
GO:0008233∼peptidase activity	10	0.05	2.04
GO:0016791∼phosphatase activity	7	0.06	2.44
GO:0004175∼endopeptidase activity	7	0.07	2.43
**KEGG pathways**	**Count**	**P-val**	**FE**
dme03010:Ribosome	3	0	30.13

Enriched GO terms (Biological Process BP, Molecular function MF and Cellular Component CC) and KEGG pathway in the set of significant probes in both comparisons (STR vs LHR and STR vs MHR). Genes count (Count), p value (P-val) and fold enrichment (FE) of significantly enriched terms are reported.

## Discussion

Gene expression analysis and evaluation of DEGs comparing oocytes before and after spawning pointed out significant results and provided a first overview on transcriptome changes that are correlated with stripped oocytes infertility. The PCA applied to the selected gene expression dataset clearly emphasized three different clusters for the 15 oocyte samples, stripped and spawned oocytes, this latter being divided into two sub-groups. When looking at these two sub-groups, the subsequent D larval rates obtained by fertilization of the remaining oocytes for each female that spawned, appeared clearly different, less than 21% for the sub-group named low hatching rate (LHR) and higher than 40% for the other one, named with medium hatching rate (MHR). These limits of D larval rates for defining oocyte quality are similar to those already considered in a proteomic-based analysis of oocyte quality in the Pacific oyster *C. gigas* [19]. Such a result urges the need of further molecular studies on oocyte quality which are actually on-going in a larger set of samples (*de Sousa J.*, unpublished data). Here the attention has been focused on the analysis of molecular signatures characterizing stripped and spawned oocytes, no matter the quality. For the sake of clarity, the most important processes that were found to have a prominent role in differentiating stripped and spawned oocytes will be discussed separately.

### Meiosis progression

Significant enrichment of BP term “Mitotic cell cycle” (FE = 2.14) clearly demonstrated that among the 198 annotated DEGs between stripped and spawned oocytes, several transcripts were implicated in the regulation of meiosis progression. Indeed, mitosis and meiosis share several steps in their respective processes and thus regulatory molecules are mostly the same in both types of cell division.

Several proteins are involved in the regulation of prophase I arrest in ovarian oocytes, the most crucial factor being the maturation promoting factor (MPF) [33]. MPF is a key G2/M phase regulator in eukaryotic cells and is composed by CDC2 kinase (known also as CDK1) and cyclin B [34]. MPF is induced during meiosis resumption and its activity is regulated by phosphorylation of CDC2 kinase. CDC2 kinase is activated when M-phase inducer phosphatase (CDC25) dephosphorylate threonin-14 and tyrosine-15 sites [35]–[37], leading, in turn, to MPF activation and meiosis resumption. In this study, a total of six probes coding for CDC25 or CDC25-like proteins was reported to be more abundant in released oocytes, suggesting a prominent role of these phosphatases in the resumption of meiotic cell cycle progression in *V. decussata*. The controlling function of CDC25 phosphatases in the meiosis I progression has been proposed in a wide range of species e.g. [38]–[41]. In mouse, both *CDC25A* and *CDC25B* were demonstrated to be critical for meiotic maturation and metaphase I spindle formation in oocytes [42]. *CDC25B*−/− knockout female mice are sterile because their oocytes cannot exit developmental arrest at meiosis prophase 1 [43], whereas *CDC25A*−/− mice exhibit early embryonic lethality [44], indicating that both proteins are required for the control of oocyte meiotic cell cycle and embryonic mitotic cell cycle, respectively. Interestingly, when injected into *Xenopus laevis* oocytes, mRNAs encoding frog homologs of mammalian CDC25A and CDC25C can induce nuclear envelope breakdown [45]. In *C. gigas* genome (http://oysterdb.cn/) seven *CDC25* genes are reported, while in the European clam the number of *CDC25* isoforms is still unknown. However the findings reported here suggest that in *V. decussata* CDC25 family members may be crucial for both control and induction of meiotic maturation.

The importance of cell-cycle proteins was also suggested by the observed expression levels of *CDC20*, which is implicated in meiosis regulation, being the activating subunit of the anaphase-promoting complex/cyclosome (APC/C). CDC20 initiates sister-chromatid separation by ordering the destruction of two key anaphase inhibitors, cyclin B1 and securin, at the transition from metaphase to anaphase, much like mitosis in somatic cells [46]. Despite SAM analysis did not show any significant variations between the two experimental groups, an ad hoc T-test, that allows estimating differences without doing any kind of correction (e.g. FDR), demonstrated that two probes encoding a CDC20-like protein were significantly more expressed in thermally-induced released oocytes (data not shown), thus suggesting an important role of this transcript in determining oocyte competence on fertilization. Notably, in mice it has been reported that CDC20 may be required for anaphase onset during the first meiosis, thus raising also the possibility that CDC20 insufficiency may be a cause of infertility in otherwise healthy females [47]–[48]. Likewise, during bovine oocyte maturation, *CDC20* down-regulation significantly reduced the rate of first polar body emission [49]. In invertebrates the role of CDC20 has not been studied so far except for *Drosophila*. In fact, fruit-fly cortex, a member of gene *CDC20*/fizzy protein family, was found to be required to complete oocyte meiosis and cooperates with CDC20 in cyclin destruction and anaphase progression in meiosis I and II [50]–[51]. The putative functioning of CDC20 has not been explored in molluscan oocytes and the over-expression of this transcript in released clam oocytes hints its importance to allow meiosis progression after fertilization.

### WNT signalling pathway

A crucial role in the molecular processes that differentiate stripped and spawned oocytes could also be proposed for a few members of the WNT signalling pathway (GO:0016055), which plays a crucial function in controlling genetic programs of embryonic development and adult homeostasis [52]. Recently, in mammals WNT pathway signalling has been implicated in ovarian development, oogenesis, and early development. Multiple WNT signalling pathway genes are expressed in mouse oocytes and pre-implantation stage embryos, as revealed by microarray analyses [53]–[54], and this has led to the hypothesis that WNTs may function in early cell fate determination events [53]. However, other studies indicate that WNT signalling pathways are likely not functional in the early embryo [55], raising the possibility that expression of WNT signalling genes in oocytes and early embryos are most likely related to functions in oogenesis (e.g., oocyte growth or maturation) [56]. In the present study the expression levels of three probes putatively encoding a WNT proteins receptor, frizzled 8 (FZD8), were more abundant in released oocytes. Such evidence might suggest that *FZD8* expression supports WNT signalling pathway activation by favouring the recognition of WNT proteins (Figure 3). Moreover, a putative ortholog of oyster *WNT4* was less abundant in female gametes extracted from mature gonads, in comparison with released oocytes. Notably, mice null for *WNT4* exhibit sex reversal and a reduced number of oocytes in new-born ovaries [57]. In the same species, ovaries of *WNT4*-mutant females were characterized by a scarce amount of oocytes, which were in the process of degenerating [58], and 80% of *WNT4* deficient germ cells failed to enter meiosis [59]. Finally a putative FJ encoding transcript was significantly (FDR<1.5%) more expressed in spawned than in stripped oocytes (Figure 3). The role of FJ has been poorly investigated in both vertebrates and invertebrates. However, a few studies focusing on FJ have been performed in *D. melanogaster*, where it has been demonstrated that this protein directly interacts with WNT4 and they act synergistically to induce planar polarity during early development [60]–[61]. Considering the lack of functional information concerning FJ, it is difficult to propose a specific role of this gene in *V. decussata*. Nevertheless it can be suggested that its expression in oocyte is probably linked to oocytes competence on fertilization.

Moreover, expression levels of a further gene involved in the regulation of WNT-mediated signal, the dishevelled segment polarity protein 3 (*DVL3*), provided additional information to elucidate the role of this pathway in clam oocytes. Ad hoc T-test (pval<0.05) demonstrated that a putative *DVL3* transcript was higher in released oocytes, suggesting the importance of this molecule in bivalve oocyte maturation. The protein encoded by *DVL3* gene is implicated in transduction signal (Figure 3) conveyed by various WNT genes and, in *Xenopus* oocytes, its expression has been reported to be extremely important for transduction of canonical WNT signals after fertilization [62]. To conclude, the hypothesis that in *V. decussata* oocytes the amount of mRNAs encoding proteins involved in WNT signalling could be extremely important to complete oocyte maturation and fertilization is a matter of concern for future investigations.

### Sex steroids and vitellogenin

In the present study, changes in mRNA levels were reported also for putative *PAQR3* and *CYP450-C17*, which were both more abundant in ovarian oocytes. Progestin and adipoQ receptor family is a group of G protein-coupled receptors including membrane progesterone receptors (mPRs) that mediate a variety of rapid cell surface-initiated progesterone actions in the reproductive system involving activation of intracellular signalling pathways. While cytochrome P450-C17 is an enzyme involved in the synthesis of 17β-estradiol (E2) during steroidogenesis. Despite in invertebrates there have been conflicting lines of evidence concerning the existence of enzymes necessary to synthesize vertebrate steroids and related nuclear receptors [63], several studies suggested a role of E2 and progesterone in gonadal development, oocytes maturation and spawning in several bivalve species [64]–[71]. Consistent with these studies, here it was demonstrated that significant variations of transcripts involved in sex steroids synthesis and activity occur during *V. decussata* oocytes maturation. In particular higher levels of *PAQR3* and *CYP450-C17* mRNAs in ovarian oocytes may be associated to higher E2 synthesis and progesterone activity in comparison with released oocytes. These data let us thinking that maturation stimuli by sex steroids are especially higher before the spawning event and most probably take part to the molecular processes inducing oocytes emission and restarting of meiosis in *V. decussata*. In agreement with such hypothesis, previous studies indicate that sex steroids have a pivotal role in the pre-spawning stage since they have stimulatory effects on gamete release in *Patinopecten yessoensis* and *Placopecten magellanicus* [71]–[73].

Another interesting finding was the significant change in expression of a putative vitellogenin (*VG*) transcript, whose expression was over the threshold of fluorescence (see section 2.3) only in released oocytes. Vitellogenins are the major precursors of egg-yolk proteins, vitellins (VNs). Vns, stored in developing oocytes, are required for oocyte growth and maturation [74]–[75] and traditionally regarded as the energy reserve for nourishment of developing embryos [64], [76]–[77]. To date, a full length sequence characterization of *VG* has been provided only for a few fish [78]–[79] and crustacean species [80]–[81]. In addition, compared with its extensive research in adults, data on *VG* expression during development are insufficient and limited to some insects and crustaceans [82]–[84]. In molluscs, *VG* expression levels were mainly evaluated in the gonadal tissue [65]–[67], [85]–[87]. In bivalve molluscs there is no direct evidence on VG synthesis, and it is still unclear whether the synthesis of a major yolk protein occurs in oocytes (auto-synthesis) [88]–[89] or in auxiliary cells (hetero-synthesis) [65]–[67], [85], [90]. The fact that in the present study *VG* mRNA was detected only in the spawned oocytes, favours the hypothesis that in *V. decussata* VG is synthesized through an auto-synthetic pathway. Moreover, based on *de Sousa* et al. [26], who reported a high expression of this *VG* transcript through all gonadal stages with mRNA increase over the time course of the gametogenesis, we could legitimately suppose that in this species also a hetero-synthesis occurs. Most probably exogenously-synthetized VG protein is transferred into oocytes, where it functions as energy reserve.

Notwithstanding the fact that the importance of VG as energy reserve has been demonstrated mainly at a protein level, *VG* mRNA stores in released oocytes could play a pivotal role, probably by providing a stock of transcripts ready to be translated into functional protein. Thus it can be hypothesized that these reserves may be crucial for released oocytes viability. Besides playing a role in the formation of yolk protein during early development, *VG* mRNA in oocytes might perform pleiotropic functions, including immune defence reactions [91]–[94]. Likewise, *VG* transcript, detected only in fertilizable released oocytes, might be an important resource in oocytes of *V. decussata* since it could be translated as a maternal factor and then possibly providing a sort of immune defence prior to embryonic gene activation and during early embryonic development. The absence of *VG* mRNA in stripped oocytes indicates that *VG* transcription in clam oocyte initiates after ovulation. Similar results have been recently obtained by *Li* and co-workers [95], who demonstrated that, in *Chlamys farreri*, *VG* mRNA is expressed during early development and that the highest levels occur in released oocytes and fertilized eggs.

Anyhow, since the site of VG synthesis in *V. decussata* remains to be further clarified and major regulations likely occur at the protein level, it is premature to draw general conclusions about the role of *VG* in released oocytes.

### mRNA processing

The mechanism supposedly involved in the retention of *VG* mRNA requires the activity of RNA-binding proteins, which are known to function as translational repressors in the cytoplasm of several eukaryotic cells [96]–[97]. This mechanism is considered to be crucial in oocytes since, during oogenesis, maternal mRNAs are synthesised and stored in a translationally dormant form and are activated either upon re-entry into meiotic division or after fertilisation [23]–[24]. During oocyte maturation and early embryogenesis in *Xenopus*, translin, a RNA-binding protein, was demonstrated to play a major role to repress maternal mRNA translation [98]. Notably in the present study, a translin-associated protein X, which is involved in nuclear transport of translin in mice [99]–[100], was expressed in all oocytes conditions and was particularly abundant in stripped oocytes. The relevance of mRNA translation in oocytes was demonstrated also by the significant variation reported for a putative homolog of the *Xenopus* oocyte zinc finger protein XlCOF28, whose mRNA amount was more abundant in spawned oocytes. The *Xenopus* zinc finger protein XlCOF28 belongs to the family of C2H2 zinc finger proteins, which are known to function as RNA-binding molecules [101]. Noteworthy, it has been recently reported that proteins belonging to this family are required for regulation of maternally supplied mRNAs during oogenesis, oocyte to embryo transition, and early embryogenesis in both vertebrates and invertebrates [102]–[103]. Based on these multiple lines of evidence, it can be hypothesized that C2H2 zinc finger proteins take part to the mechanisms that regulate mRNA silencing in *V. decussata* oocytes.

### Calcium regulation

A further interesting result concerns the differential expression of several transcripts involved in calcium (Ca2+) signalling. Calcium ions are the most common second messengers in animal cells [104] and their transient elevations regulate numerous cell functions. There has been a long-standing debate as to whether Ca2+ signals are required for oocyte meiosis and numerous conflicting studies argue that the relationship between Ca2+ and oocyte maturation is complex, possibly due to fine regulation *via* very constrained levels of Ca2+ [20], [105]. The role that external calcium, through voltage-gated channels, might play in the induction of GVBD was first reported in molluscs that are both fertilized at the PI stage [14], [106]–[107], or undergo the second arrest in MI [3], [108]. It was soon recognized that also calcium influx from intracellular storages plays a crucial role in almost all species studied independently from their peculiar meiotic arrest [8], [107], [109]. In particular, the interplay between external and internal calcium currents is evident in *Venerupis*, where a serotonin-induced surge of intracellular calcium was shown to trigger maturation even in the absence of external calcium [8]. In the present study, the differences in the amount of mRNA encoding putative calcium-activated chloride channel regulator 4 was an additional clue for assessing that the regulation of intracellular Ca2+ plays an important role in *V. decussata* oocytes maturation. Likewise, a putative sodium/calcium exchanger 3 (*NCX3*) was reported to be more abundant in released oocytes. The Na+/Ca2+ exchanger proteins represent an antiporter system that utilizes the electrochemical gradient of Na+ to catalyze Ca2+ extrusion from the cytosol or organelle matrix. NCX proteins explore the electrochemical gradient of Na+ to mediate Ca2+ fluxes in exchange with Na+ either in the Ca2+ efflux (forward) or Ca2+ influx (reverse) mode, whereas the directionality depends on ionic concentrations and membrane potential [110]. Despite NCX is considered one of the most important cellular mechanisms for removing Ca2+, it should be noted that under special circumstances and in some types of cells, including oocytes, the transporter might operate in the “Ca2+ entry” mode and play a critical role in mediating calcium influx rather than efflux [111]. Accordingly to the reverse mode, the higher mRNA levels of a putative *NCX* reported in clam released oocytes (compared to stripped oocytes) could be an important requisite to allow Ca2+ influx, thus favouring release from metaphase I and fertilization. Despite in pig oocytes it has been demonstrated that Ca2+ entry through a Na+/Ca2+ exchanger did not induce meiotic resumption from metaphase II [112], the role of this antiporter system in bivalves has not been studied yet and we cannot exclude that in other species it can act differently. Moreover, even if we suppose a forward mode (Ca2+ efflux) of NCX, the relevance of this system does not became secondary, since intracellular calcium variations are key regulators affecting oocytes development and fertilization. Therefore, to evaluate NCX functioning in *V. decussata* and confirm its importance during oocyte maturation, more specific studies are needed.

Conversely, homologs of *C. gigas* regucalcin and calmodulin showed higher expression levels in ovarian oocytes. Calmodulin and regucalcin are calcium-binding proteins that are supposed to contribute to meiosis regulation [113]–[114] and their mRNA variation suggests an involvement in maintaining calcium homeostasis in immature oocytes. Notably, regucalcin might function as transcriptional regulator and its over-expression in NRK52E cells was demonstrated to repress the expression of L-type Ca2+ channel and Ca2+-sensing receptors [115]. This evidence is of particular interest since L-type Ca2+ channels are thought to be specifically involved in meiosis re-initiation [20]. Indeed, a high level of regucalcin in stripped oocytes might be implicated in maintaining meiosis blocked at prophase I stage, thus hampering fecundation by sperm. Despite little is known about the molecular regulation of intracellular Ca2+ occurring during oocytes maturation in bivalves, these preliminary results pointed out a few important genes possibly involved in such a complex mechanism.

### Ceramide metabolism

Finally, another metabolic process potentially implicated in oocyte maturation in *V. decussata* was the regulation of ceramide levels. Ceramide is a signal sphingolipid thought to influence oocyte maturation and quality. The enzymes controlling the metabolism of ceramide in oocytes have been poorly studied in molluscs and only recently sequences of genes associated with ceramide metabolism and signalling have been investigated in the Pacific oyster [116]. Conversely, in vertebrates quite a few studies have been focused on the role of ceramide in oocytes and two main hypotheses have been suggested [117]. First, it has been proposed that the generation of ceramide is part of the signal transduction pathway activated in response to progesterone, and that its increase is likely functionally important in the resumption of meiotic cycle [118]–[120]. Second, it has been recently demonstrated that ceramide induces default apoptosis in oocytes and has a central role in age-related decrease of egg quality [121]–[122]. In the present study, at least three enzymes involved in ceramide metabolism were found to be differentially expressed between stripped and spawned oocytes. A ceramide synthase, less abundant in ovarian oocytes, a ceramidase B and a sphingomyelinase, both less abundant in released oocytes. Since no data are available on normal ceramide homeostasis in bivalve oocytes, a comprehensive interpretation of the reported mRNA changes is particularly challenging in association with oocytes maturation and competence development.

## Conclusions

To conclude, gene expression analysis allowed the identification of important mechanisms that could have a key role in the process of bivalve oocyte development. Differences in mRNA expression of some important genes have been detected providing a more comprehensive picture of key processes affecting *V. decussata* oocyte maturation and competence acquisition. Noteworthy, transcripts for which the expression level was subjected to significant changes after spawning were those encoding proteins involved in cell cycle progression, calcium regulation and WNT signalling. These biological processes most likely play a major role in female gamete maturation and competence acquisition. While suggestive, the obtained results would require further validation through experimental manipulation of the highlighted signalling pathways. In perspective, functional studies could represent an interesting field to be explored. Gene functions are primarily assessed on the basis of altered phenotypes associated with gene disruption or protein inactivation. Recent studies carried out in *C. gigas*, demonstrated that the in vivo functional inactivation of proteins through polyclonal antibody injection [123] or RNA interference [124]–[125] could provide important information to unravel gene functions in bivalves. Nonetheless, pharmacological stimulators and disruptors, inferred from the experience achieved in most studied vertebrate species, can be employed to address the role of a gene and chiefly test for improvement of hatchery practices.

## Supporting Information

Table S1 Principal Component 1 and Principal Component 2 highest eigenvector values. The table lists the 1,593 probes (5% out of the total amount of probes) with the highest eigenvector values in both PC1 and PC2. The eigenvector values and the putative annotation against UniProtKB/SwissProt database are also reported.
(XLSX)

Table S2 Significance Analysis of Microarray (SAM). List of annotated probes that were differentially expressed (FDR <1.5%; FC>1.5) in both the two comparisons LHR vs STR oocytes and MHR vs STR oocytes with the corresponding fold change value. Shared significant probes between the two comparisons were also summarized. Colors identify transcripts more abundant in spawned (green) and stripped oocytes (red). Putative annotation against UniProtK/SwissProt and Pacific oyster databases of each probe are specified.
(XLSX)
